# A mixed-methods study of depressive-symptom severity among Thai university students and the underestimated role of social media

**DOI:** 10.3389/frcha.2026.1907727

**Published:** 2026-10-15

**Authors:** Pichit Boonkrong, Kodchakorn Khunboonjun, Subij Shakya, Junwei Yang, Naphatsakon Vitoonmetha, Manaporn Chatchumni

**Affiliations:** 1 College of Biomedical Engineering, Rangsit University, Pathum Thani, Thailand; 2 Department of Food and Nutrition, University of Helsinki, Helsinki, Finland; 3 Department of Electrical Engineering, Faculty of Engineering, Chulalongkorn University, Bangkok, Thailand; 4 Health Welfare Office, Rangsit University, Pathum Thani, Thailand; 5 School of Nursing, Rangsit University, Pathum Thani, Thailand

**Keywords:** depressive symptoms, health promotion, psychological distress, social media, social support

## Abstract

**Purpose:**

University students face academic, social, economic, and digital stressors. Social-media use may be under-recognized as a correlate of depressive symptoms because online engagement is often viewed as routine communication, leisure, and academic activity. This study examined factors associated with depressive-symptom severity among Thai university students and explored the mismatch between perceived stressors and the observed association with social-media use.

**Methods:**

A cross-sectional mixed-methods study was conducted among 789 undergraduates at Rangsit University, Thailand. Quantitative data were collected using a structured questionnaire and the Center for Epidemiologic Studies Depression Scale (CES-D), categorized as low symptoms (0–15), mild-to-moderate (16–26), or severe (27–60) symptoms. Associations were examined using *χ*^2^ tests and ordinal logistic regression. A purposive subsample of 12 students participated in semi-structured interviews on social-media routines, online comparison, nighttime use and sleep, coping, social support, and help-seeking. All interviews were audio-recorded and supplemented by contemporaneous field notes. Qualitative data were analyzed thematically and integrated with quantitative findings through joint comparison.

**Results:**

Clinically significant depressive symptoms (CES-D ≥ 16) were reported by 49.68% of students. Academic year, major, GPAX, primary source of personal support, and daily social-media use were independently associated with symptom severity. Compared with students using social media >3 h/day, those using it <1 h/day (OR = 0.125, 95% CI: 0.040–0.391) or 1–3 h/day (OR = 0.424, 95% CI: 0.278–0.647) had lower odds of greater symptom severity. Internet/social-media use ranked only fifth among eight perceived stressors (37.01% agreement). Three qualitative themes described social media as an embedded and ambivalent part of student life, psychological effects shaped by patterns and interpretations of use, and distress managed mainly through self-coping and trusted informal support.

**Conclusion:**

The key mixed-methods finding was a perception–association mismatch: social-media use was not perceived as a leading stressor yet showed the strongest adjusted association with depressive-symptom severity among the factors examined. These cross-sectional findings do not establish causality but support digital-wellbeing assessment beyond duration alone.

## 1 Scope statement

This manuscript fits the scope of Frontiers in Child and Adolescent Psychiatry, particularly the specialty section Adolescent Mental Health and Interventions, because it examines depressive-symptom severity and modifiable psychosocial and digital correlates among university students spanning late adolescence and the transition to emerging adulthood. By integrating CES-D screening, ordinal logistic regression, perceived-stressor ratings, and qualitative interviews, the study addresses digital wellbeing, social support, help-seeking, and early identification of depressive symptoms in a developmental period relevant to prevention and transitional-age mental-health care. The study is exploratory and does not infer causal effects from the cross-sectional associations.

## 2 Introduction

Depression among university students has emerged as a significant public health concern globally. University students are exposed to multiple stressors, including academic pressure, financial difficulties, social transitions, and lifestyle disruptions, all of which may heighten vulnerability to depressive symptoms (1–4). Such symptoms have been associated with reduced academic performance, increased dropout rates, and impaired quality of life. The Center for Epidemiologic Studies Depression Scale (CES-D) has been widely validated as a screening tool for depressive symptoms in community and student populations (5–8). Early identification of depressive symptoms and their associated factors is therefore essential for designing effective, targeted mental health interventions within higher education settings.

Despite growing recognition of student mental health as a priority, existing evidence on depressive symptoms among university students in Southeast Asia remains limited and fragmented. Most studies have been conducted in Western, high-income contexts, where cultural, economic, and educational systems differ substantially from those in ASEAN member states (2, 4, 5). Consequently, the generalizability of findings from these settings to Thai or broader ASEAN university populations is uncertain (2, 4, 5, 9). Furthermore, among the few studies available in the region, several methodological limitations persist. Many have employed small sample sizes, convenience sampling strategies, or non-validated screening instruments, thereby limiting the precision and comparability of prevalence estimates. Others have focused narrowly on single domains of associated factor, e.g., academic stress or sleep quality (10–13). They have not concurrently examined the multidimensional nature of depressive symptoms, which includes socio-demographic, academic, lifestyle, and social support variables within a single analytical framework. Additionally, the role of mental health literacy has been underexplored as a modifiable factor associated with depressive symptoms among Asian university students. Mental health literacy is defined as knowledge and beliefs about mental disorders that aid recognition, management, or prevention (11, 14).

Although all participants were undergraduate students aged 18 years or older, the sample remains relevant to a child and adolescent mental-health readership because most participants were aged 18–20 years, spanning late adolescence and the transition to emerging adulthood (15, 16). This developmental period involves continuing identity formation, increasing independence, changing peer and family relationships, and sustained sensitivity to digital environments (17–20). Evidence from this age group may therefore inform continuity in mental-health prevention and early intervention as young people transition from adolescent-oriented services to adult systems of care. Social media is deeply embedded in Gen Z students’ academic communication, peer relationships, entertainment, and self-presentation. Supportive online interaction may promote connection, whereas prolonged passive use, upward social comparison, fear of missing out, and nighttime engagement may be associated with poorer sleep and greater psychological distress (3, 12, 18, 21). Because online engagement is often normalized as leisure, connection, or academic participation, students may not identify it as a salient source of stress even when usage patterns are statistically associated with depressive-symptom severity. This perception–association mismatch provides the rationale for examining the potentially underestimated role of social media.

Under the ASEAN University Network–Health Promotion Network (AUN-HPN) framework at Rangsit University, Thailand, this mixed-methods cross-sectional study addressed these gaps. Rather than treating social media solely as “screen time,” the study examined whether its statistical association with depressive-symptom severity corresponded with students’ own appraisal of internet/social-media use as a stressor (22, 23). The objectives were to examine depressive-symptom severity among Thai undergraduate students; identify sociodemographic, academic, lifestyle, social-support, and social-media factors associated with severity; compare students’ perceived stressors with observed statistical associations; and use qualitative accounts to explain convergence or divergence between these two forms of evidence.

## 3 Methods

This section describes the study design, setting, participants, eligibility criteria, variables, measurement instruments, sample-size determination, and analytical methods. Reporting of the quantitative component was guided by the STROBE statement for cross-sectional studies, whereas reporting of the qualitative component was informed by the Consolidated Criteria for Reporting Qualitative Research (COREQ) (24, 25). The methodology followed a mixed-methods design integrating quantitative survey data (CES-D and perceived stress) with qualitative interviews as visualized in Figure 1.

**Figure 1:**
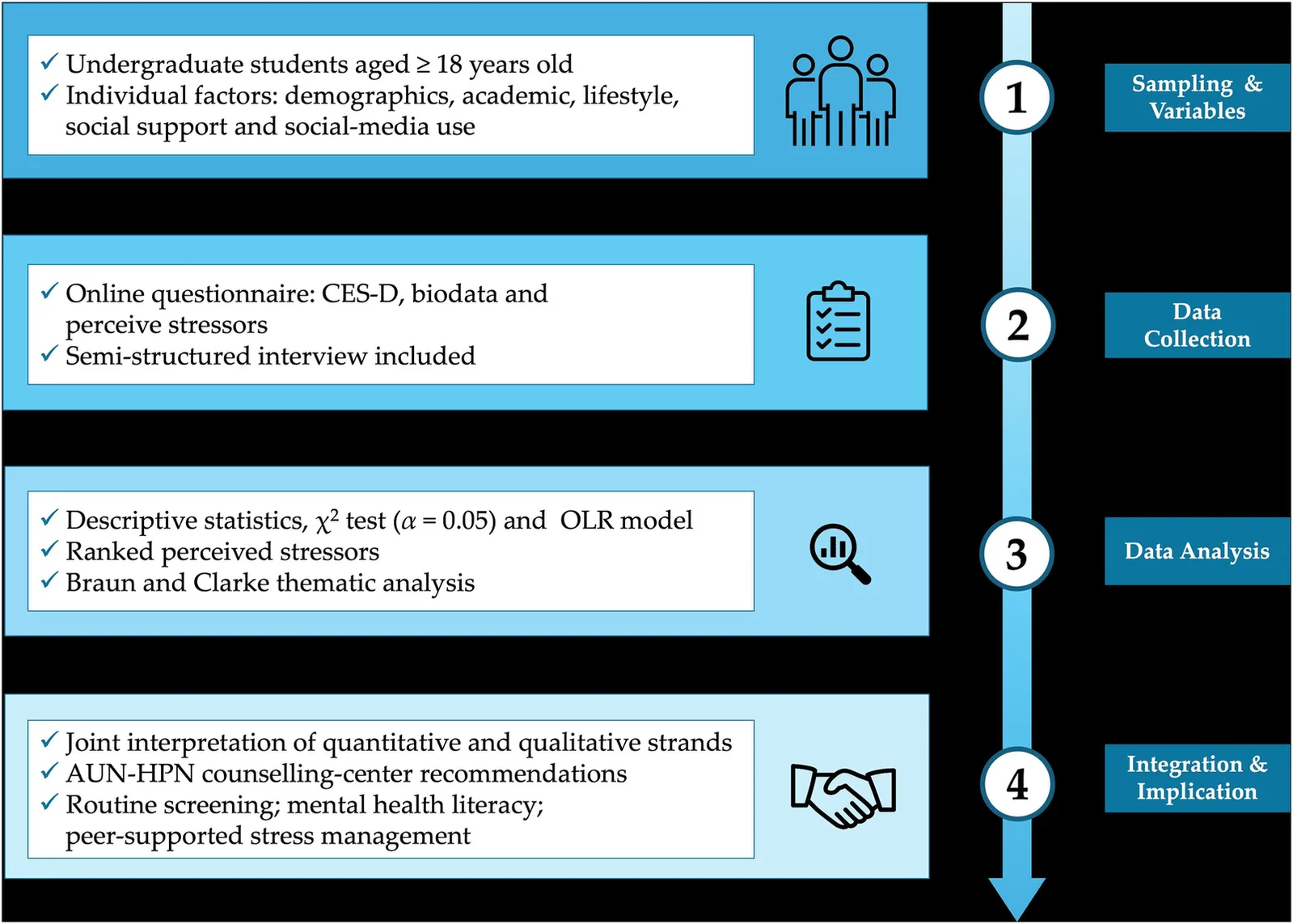
Conceptual framework of factors independently associated with depressive-symptom severity among university students.

### 3.1 Ethics statement

The Rangsit University Ethics Review Board approved the study (COA No. RSUERB2024-142). Before accessing the online questionnaire, each participant reviewed an electronic participant information sheet and provided electronic informed consent. Participation was voluntary, and participants could discontinue the questionnaire at any time without penalty. Survey responses were collected without direct personal identifiers and were stored securely, with access restricted to the research team. Data collection and management were conducted in accordance with Thailand's Personal Data Protection Act. Because all eligible participants were aged 18 years or older, parental consent was not required.

### 3.2 Study design and setting

A cross-sectional, mixed-methods design was employed at Rangsit University, a private university in Pathum Thani Province, Thailand, combining a quantitative survey with a qualitative interview component. In the quantitative phase, 789 undergraduate students completed the Center for Epidemiologic Studies Depression Scale (CES-D) and a structured questionnaire capturing socio-demographic, academic, lifestyle, and social-support variables. In the qualitative phase, semi-structured follow-up interviews were conducted with a purposively selected subset of participants to explore the psychosocial circumstances underlying their depressive symptoms. Integrating the two strands allowed the statistical patterns to be interpreted alongside students’ lived experiences. Data were collected during the second semester of the 2024–2025 academic year. The findings are intended to inform targeted mental health promotion and health literacy programs for university students.

### 3.3 Participants

The quantitative survey used convenience sampling. The online questionnaire was distributed through university email and official student networks to currently enrolled undergraduates who voluntarily chose to participate. After reviewing the electronic participant-information sheet and providing electronic informed consent, participants completed the biodata section, CES-D, and perceived-stressor items. Participation was voluntary, and participants could withdraw without penalty. No course credit or financial incentive was provided. A purposive subsample was subsequently invited for semi-structured follow-up interviews. No educational intervention was implemented during the study period.

#### 3.3.1 Eligibility criteria

Undergraduate students enrolled at Rangsit University during the study period were eligible if they were (1) aged ≥18 years, (2) actively enrolled in any faculty or academic year, and (3) able to read and complete the Thai-language study instruments. Students were excluded if they were younger than 18 years, were not currently enrolled, declined consent, submitted an incomplete questionnaire, or provided a duplicate response. No upper age limit was specified. In the analyzed sample, participants ranged from 18 to 27 years of age.

#### 3.3.2 Sample size

Participants were recruited through convenience sampling. The minimum required sample was estimated using Cochran's formula for a single proportion, $n_{0}\;=\;Z^{2}p(1-p)/e^{2}$, with *Z*  = 1.96, *p*  = 0.5 (the most conservative assumption), and *e*  = 0.05, yielding 385 participants (26–28). Because the undergraduate population is finite, the finite-population correction only reduces this figure, so 385 represents a conservative lower bound. The achieved sample of 789 substantially exceeded this threshold; at the observed prevalence of clinically significant symptoms, the realized margin of error was approximately ±3.3% at 95% confidence, tighter than the conventional ±5%.

### 3.4 Measures and instruments

A semi-structured questionnaire was employed to collect data on depressive symptoms, socio-demographic and academic characteristics, lifestyle behaviors, social support, and perceived sources of stress. Depressive symptoms, as the primary outcome, were measured using a validated screening instrument.

#### 3.4.1 Depressive symptoms

Depressive symptoms were assessed with the 20-item Center for Epidemiologic Studies Depression Scale (CES-D), scored from 0 to 60, with higher scores indicating greater symptom severity (5, 29, 30). The Thai-language CES-D has previously undergone psychometric evaluation in Thai populations (31). In the present sample, internal consistency was acceptable (Cronbach's *α* = 0.81). For ordinal logistic regression (OLR), total scores were classified into three ordered categories: low symptoms (0–15), mild-to-moderate symptoms (16–26), and severe symptoms (27–60). The CES-D was used as a screening measure rather than a diagnostic instrument.

#### 3.4.2 Independent variables and perceived stressors

The 13 independent variables were drawn from the self-reported biodata section: sex, age group, religion, hometown region, academic year, major of study, cumulative grade point average (GPAX), smoking status, alcohol use, presence of comorbidity, relationship status, primary source of personal support, and daily social-media use. Participants also rated eight perceived stress domains on a four-point agreement scale (1 = strongly disagree to 4 = strongly agree): ethnicity and culture; language barrier; educational system; relationship and social environment; health conditions/comorbidity; genetics/gender; financial and economic status; and internet/social-media usage. For Table 3, Agreement (%) represents the percentage selecting “agree” or “strongly agree” (scores 3 or 4) for the corresponding domain; rankings are based on mean item ratings rather than individual rank-order choices.

#### 3.4.3 Qualitative sampling and interview procedures

Semi-structured follow-up interviews were conducted with 12 purposively selected survey participants to obtain variation across academic backgrounds and CES-D symptom-severity levels. The qualitative subsample included six female and six male students aged 18–26 years and represented both health-science and social-science-related disciplines at Rangsit University, including Health Sciences, Engineering and Technology, Arts and Business, and Humanities and Social Sciences. All 12 enrolled qualitative participants completed the interview; no drop-outs occurred. Interviews were conducted in the meeting room of the Health Welfare Office, Rangsit University. All 12 interviews were audio-recorded with participants’ consent, and contemporaneous field notes were also taken. No repeat interviews with the same participants were conducted. The interview guide used four broad, open-ended topic areas: (1) everyday social-media routines, including timing, purpose, and feelings before, during, and after use; (2) online social comparison and its influence on self-perception and emotional wellbeing; (3) nighttime social-media use and perceived effects on sleep, fatigue, mood, concentration, and next-day functioning; and (4) coping with emotional distress, preferred sources of support, and perceived barriers to professional help-seeking. The questions served as prompts for data collection rather than predetermined analytic themes. Participants were encouraged to elaborate on experiences they considered meaningful, and follow-up probes were used to explore both adverse and neutral or beneficial experiences. Interviews continued until no substantially new interpretive content emerged after the tenth interview; two additional participant interviews were conducted to assess thematic consistency. The retained study documentation did not contain sufficiently reliable information on interviewer demographic/training characteristics, the exact number of students initially approached or declining before enrollment, the precise mode of initial contact, interview duration, presence of non-participants, or participant/member checking. These items are therefore reported transparently as unavailable rather than reconstructed retrospectively in the accompanying COREQ checklist.

#### 3.4.4 Instrument validity and reliability

Content validity of the study-developed perceived-stressor items and semi-structured interview guide was evaluated by four clinical psychologists using the Index of Item-Objective Congruence (IOC); items with an IOC value ≥0.50 were retained. The Thai-language CES-D was an existing instrument with prior psychometric evaluation in Thai populations (31) and was not revalidated through the IOC procedure. In the present sample, internal consistency was acceptable for the CES-D (Cronbach's *α* = 0.81) and high for the eight perceived-stressor items (Cronbach's *α* = 0.86). The interview guide was reviewed for content relevance and clarity and was pilot-tested before use.

### 3.5 Mixed-methods analytical approach

Quantitative data were analyzed using descriptive statistics, *χ*^2^ tests, and OLR to identify factors associated with depressive-symptom severity. Qualitative data from semi-structured interviews were analyzed using thematic analysis to explore students’ lived experiences. Findings from both strands were then compared and interpreted jointly to provide a more comprehensive understanding.

#### 3.5.1 Quantitative analysis

Analyses were performed in *Jamovi* (version 2.3.28). Descriptive statistics summarized participant characteristics and the perceived-stress items (32). For ordinal analysis, CES-D scores were classified into three ordered categories: low symptoms (0–15), mild-to-moderate (16–26), and severe (27–60). Bivariate associations between each independent variable and the three-level CES-D outcome were examined using Pearson *χ*^2^ tests. To identify factors independently associated with greater severity, ordinal (cumulative-logit, proportional-odds) logistic regression was conducted with the ordered CES-D categories as the dependent variable; variables significant at the bivariate level (*p* < 0.05) were entered. Mathematically, the OLR model (cumulative logit, proportional odds) was specified as

$$\log\left[\frac{P(\mathrm{CES}-D\leq j)}{P(\mathrm{CES}-D>j)}\right]=\gamma_{j}-(\beta_{1}X_{1}+\ldots +\beta_{k}X_{k}),\;\;j=1,2.$$

where $\gamma_{j}$ are the threshold parameters for the two cumulative logits (low symptoms vs. higher severity; low/mild-to-moderate vs. severe). The linear predictors are encoded as dummy-coded categorical variables. Under the proportional odds assumption, each predictor's coefficient $\beta_{i}$ is constant across both cumulative logits, yielding a single odds ratio per variable: $\mathrm{OR}=\exp(\beta_{i})$ for moving from a lower to a higher CES-D severity level. Adjusted odds ratios with 95% confidence intervals were reported, and the proportional-odds assumption was checked using the test of parallel lines, with a partial proportional-odds model considered if violated. Statistical significance was set *α* = 0.05 (two-tailed).

#### 3.5.2 Qualitative analysis

Qualitative data were analyzed using Braun and Clarke's thematic-analysis framework (33, 34). Three researchers independently familiarized themselves with the qualitative material, including the audio-recorded interviews and contemporaneous field notes, and generated initial codes from meaningful units across interviews. Coding was data-led within the study objectives: the four interview topic areas organized data collection but were not treated as *a priori* themes, and codes could occur within or across topic areas. The researchers compared and consolidated related codes, developed candidate themes, reviewed themes against the qualitative material, and refined theme definitions through discussion. Divergent or negative cases were retained rather than excluded when they challenged the dominant pattern. Disagreements were resolved through discussion and consensus, and an audit trail documented coding decisions and theme revisions. Full verbatim transcripts were not prepared; selected short quotations were reported only where wording could be confirmed from the recorded interview material and field notes. No dedicated qualitative-analysis software was documented in the retained study records.

#### 3.5.3 Mixed-methods integration

Integration occurred after the quantitative and qualitative strands were analyzed separately, consistent with a convergent mixed-methods design (35, 36). Key quantitative results, including CES-D symptom-severity patterns, adjusted ordinal-regression associations, and perceived-stressor rankings, were aligned with qualitative codes and themes in a joint comparison matrix. Relationships between strands were considered as convergence, complementarity, or discordance. Particular attention was given to the discrepancy between the relatively low perceived ranking of internet/social-media use and its comparatively strong adjusted association with CES-D severity. Qualitative findings were used to contextualize, rather than causally explain, the quantitative associations by examining how timing, purpose, comparison processes, sleep-related use, self-regulation, coping, and help-seeking shaped students’ experiences.

## 4 Results

This section first describes participant characteristics and the prevalence of clinically significant depressive symptoms (CES-D ≥ 16). It then presents bivariate associations, ordinal logistic regression results, and students’ perceived sources of stress. Finally, qualitative themes are reported and integrated with the quantitative findings to examine the discrepancy concerning social-media use.

### 4.1 Participant characteristics and prevalence of depressive symptoms

A total of 789 undergraduate students at Rangsit University completed the survey and were included in the analysis. The mean age was 20.8 years (SD = 2.12; range 18–27 years); 497 participants were aged 18–20 years and 292 were aged 21–27 years. Most participants were female (62.86%), followed by male (29.91%) and non-binary (7.22%) students. The sample was drawn from across faculties and academic years. Depressive symptoms were assessed using the CES-D and classified into three ordered symptom-severity levels. Applying the established screening threshold of CES-D ≥ 16, 49.68% of participants (*n* = 392) reported clinically significant depressive-symptom scores, while 50.32% (*n* = 397) were in the low-symptom category (0–15). These categories describe screening severity and should not be interpreted as clinical diagnoses.

### 4.2 Bivariate associations with depressive symptoms

Bivariate associations were assessed using *χ*^2^ tests across all independent variables. Table 1 summarizes these comparisons, showing statistically significant associations for academic major, GPAX, and several other variables at the *α* = 0.05 level. Significant bivariate associations with depressive-symptom severity were observed for five factors. Academic year was significant (*χ*^2^ = 17.973, *p* = 0.006), with senior students showing the highest proportion of severe symptoms (30.53%) compared to freshmen (20.35%). Major of study was strongly associated (*χ*^2^ = 36.667, *p* < 0.001); Engineering and Technology students had the highest severe rate (35.66%), while Health Sciences students had the lowest (11.59%). GPAX also showed significant differences (*χ*^2^ = 26.246, *p* < 0.001); students with the lowest GPAX (2.00–2.49) had the highest severe symptoms (36.90%).

**Table 1:** Prevalence and comparison of CES-D scores across 13 factors (*n* = 789).

Factors	Label and Description	Counts	(0–15)	(16–26)	(27–60)	*χ* ^2^	Sig.
Sex	1: Male	236	46.19%	27.97%	25.85%		
	2: Female	496	52.62%	28.02%	19.35%		
	3: Non-Binary	57	47.37%	29.82%	22.81%	4.658	0.324
Age	1: 18–20	497	53.32%	25.96%	20.72%		
	2: 21–27	292	45.21%	31.85%	22.95%	5.099	0.078
Religion	1: Buddhist	716	51.26%	27.93%	20.81%		
	2: Christian	15	33.33%	46.67%	20.00%		
	3: Islam	34	50.00%	29.41%	20.59%		
	4: Others	24	33.33%	20.83%	45.83%	11.459	0.075
Hometown	1: Southern	111	51.35%	33.33%	15.32%		
	2: Northen	115	51.30%	26.09%	22.61%		
	3: Central/ East/ West	410	48.78%	28.29%	22.93%		
	4: Norteastern	153	52.94%	25.49%	21.57%	4.492	0.610
Year	1: Freshmen	172	53.49%	26.16%	20.35%		
	2: Sophomore	237	55.27%	25.32%	19.41%		
	3: Junior	285	50.88%	28.07%	21.05%		
	4: Senior & Higher	95	30.53%	38.95%	30.53%	17.973	**0**.**006**
Major	1: Health Sciences	276	59.78%	28.62%	11.59%		
	2: Engineering & Technology	143	39.16%	25.17%	35.66%		
	3: Art & Business	169	46.15%	30.77%	23.08%		
	4: Humanity & Social Sciences	201	48.76%	27.36%	23.88%	36.667	**< 0.001**
GPAX	1: 2.00–2.49	84	33.33%	29.76%	36.90%		
	2: 2.50–2.99	161	42.86%	34.16%	22.98%		
	3: 3.00–3.49	284	51.76%	28.52%	19.72%		
	4: 3.50–4.00	260	58.85%	23.46%	17.69%	26.246	**< 0.001**
Smoking	0: No	683	51.24%	28.26%	20.50%		
	1: Yes	106	44.34%	27.36%	28.30%	3.490	0.175
Alcohol	0: No	491	50.31%	28.11%	21.59%		
	1: Yes	298	50.34%	28.19%	21.48%	0.012	0.998
Social Media	1: < 1 h/ day	22	81.82%	13.64%	4.55%		
	2: 1–3 h/ day	120	66.67%	22.50%	10.83%		
	3: > 3 h/ day	647	46.21%	29.68%	24.11%	27.741	**< 0.001**
Comorbidity	0: No	648	52.47%	27.47%	20.06%		
	1: Yes	141	40.43%	31.21%	28.37%	7.622	**0**.**022**
Relationship	0: Single	482	51.66%	28.63%	19.71%		
	1: With Partner	307	48.21%	27.36%	24.43%	2.491	0.288
Consultant	1: Family	223	65.92%	19.73%	14.35%		
	2: Close Friends	361	46.26%	32.96%	20.78%		
	3: Partner	151	42.38%	31.13%	26.49%		
	4: Teacher and etc.	54	35.19%	22.22%	42.59%	44.330	**< 0.001**

Bold values indicate statistically significant associations (*p* < 0.05).

Daily social-media use was significantly associated (*χ*^2^ = 27.741, *p* < 0.001); heavy users (>3 h/day) reported more severe symptoms (24.11%) than light users (<1 h/day, 4.55%). Comorbidity was significant (*χ*^2^ = 7.622, *p* = 0.022), with students reporting comorbid conditions showing higher severe rates (28.37% vs. 20.06%). The primary consultant for personal problems was also significant (*χ*^2^ = 44.330, *p* < 0.001); students who consulted teachers or others had the highest severe symptoms (42.59%), while those consulting family had the lowest (14.35%). No significant associations were found for sex, age, religion, hometown, smoking, alcohol use, or relationship status (all *p* > 0.05).

### 4.3 Ordinal logistic regression results

In the OLR model, with CES-D severity (low, mild-to-moderate, and severe) as the ordered outcome, five factors were significantly associated with higher depressive-symptom levels: academic year (Wald *χ*^2^ = 14.18, *p* = 0.003), major of study (*χ*^2^ = 19.37, *p* < 0.001), GPAX level (*χ*^2^ = 22.43, *p* < 0.001), primary consultant for personal problems (*χ*^2^ = 24.29, *p* < 0.001), and daily social-media use (*χ*^2^ = 32.32, *p* < 0.001). Gender, age group, religion, hometown, smoking, alcohol use, comorbidity, and relationship status were not significant. The omnibus test indicated that the model containing these five factors fit the data better than the intercept-only model (*χ*^2^(14) = 128.12, *p* < 0.001). The Nagelkerke pseudo-R^2^ was 0.228; this index indicates improvement in model fit but should not be interpreted as the exact proportion of outcome variance explained. The AIC was 913.18 and the BIC was 987.91. Table 2 presents the cumulative-logit regression results.

**Table 2:** Ordinal logistic regression of CES-D depressive-symptom severity on retained factors (*N* = 789).

Factor and comparison category	B	SE	Wald	Sig.	OR	95% CI for OR
Thresholds						
Low | Mild–moderate	−1.395	0.379	13.576	<0.001	—	—
Mild–moderate | Severe	0.057	0.375	0.023	0.878	—	—
Academic year (ref: Year 4, Senior)						
Year 1 (Freshman)	−0.716	0.257	7.778	0.005	0.489	0.296–0.808
Year 2 (Sophomore)	−0.844	0.241	12.299	<0.001	0.430	0.268–0.689
Year 3 (Junior)	−0.834	0.235	12.633	<0.001	0.434	0.274–0.688
Major of study (ref: Major 4)						
Health Sciences	−0.590	0.188	9.864	0.002	0.554	0.384–0.801
Engineering & Technology	0.382	0.214	3.190	0.074	1.465	0.964–2.226
Humanities & Social Sciences	−0.080	0.208	0.147	0.702	0.923	0.615–1.387
GPAX level (ref: 3.50–4.00)						
2.00–2.49	1.151	0.251	21.055	<0.001	3.161	1.933–5.165
2.50–2.99	0.525	0.206	6.503	0.011	1.690	1.129–2.529
3.00–3.49	0.293	0.177	2.745	0.098	1.340	0.947–1.896
Primary consultant (ref: Other)						
Family	−1.242	0.299	17.192	<0.001	0.289	0.161–0.519
Close friends	−0.624	0.281	4.952	0.026	0.536	0.309–0.929
Partner	−0.298	0.305	0.955	0.328	0.742	0.408–1.350
Daily social-media use (ref: >3 h/day)						
< 1 h/day	−2.083	0.584	12.714	<0.001	0.125	0.040–0.391
1–3 h/day	−0.858	0.215	15.903	<0.001	0.424	0.278–0.647

Outcome = CES-D severity (low < mild-to-moderate < severe). Model fitted with PLUM, logit link; proportional-odds assumption supported by the test of parallel lines.

The two thresholds were correctly ordered (−1.395 and 0.057), supporting the ordering of the three-level outcome. Daily social-media use showed the strongest statistical association among the factors examined: relative to students reporting >3 h/day, those reporting <1 h/day (OR = 0.125, 95% CI: 0.040–0.391) or 1–3 h/day (OR = 0.424, 95% CI: 0.278–0.647) had lower odds of being in a higher depressive-symptom category. Source of personal support was also associated with severity; compared with consulting teachers or others, confiding in family (OR = 0.289, 95% CI: 0.161–0.519) or close friends (OR = 0.536, 95% CI: 0.309–0.929) was associated with lower odds. Lower academic performance was associated with higher odds of greater severity, with the lowest GPAX band (2.00–2.49) showing more than threefold higher odds than the reference band (OR = 3.161, 95% CI: 1.933–5.165). Health Sciences students had lower odds than the reference major (OR = 0.554, 95% CI: 0.384–0.801), and junior cohorts had lower odds than senior students.

### 4.4 Perceived sources of stress

Beyond the CES-D assessment, students rated eight potential sources of stress on a four-point agreement scale; the items showed high internal consistency (Cronbach's *α* = 0.86). Relationship and social environment was most strongly endorsed (mean 2.71; 56.40% agreement), followed by financial and economic status (2.62; 53.87%) and the educational system (2.54; 50.19%). Internet/social-media use ranked only fifth (37.01% agreement). In contrast, daily social-media use showed the strongest statistical association with depressive-symptom severity among the factors included in the ordinal regression model. This discordance—not the regression result alone—is a central finding: students did not perceive social media as a leading stressor even though usage duration differentiated symptom-severity categories. The result suggests that digital-wellbeing initiatives should address not only time online but also students’ awareness of the contexts and emotional experiences accompanying use. Table 3 presents student-perceived sources of stress across eight domains.

**Table 3:** Student-perceived sources of stress across eight domains, ranked by mean rating (*n* = 789).

Rank	Perceived source of stress	Mean (SD)	Median (IQR)	Agreement (%)
1	Relationship and social environment	2.71 (0.98)	3 (2–4)	56.40
2	Financial and economic status	2.62 (1.05)	3 (2–4)	53.87
3	Educational system	2.54 (0.99)	3 (2–3)	50.19
4	Health conditions/ comorbidity	2.34 (1.07)	2 (1–3)	41.44
5	Internet/social-media usage	2.19 (1.05)	2 (1–3)	37.01
6	Language barrier	1.96 (0.87)	2 (1–3)	26.49
7	Genetics/Gender	1.67 (0.87)	1 (1–2)	15.59
8	Ethnicity and culture	1.49 (0.72)	1 (1–2)	10.77

Agreement (%) = percentage selecting “agree” or “strongly agree” (scores 3 or 4) for the corresponding stress domain. Rankings are based on mean item ratings and do not represent the percentage assigning a domain a specific rank.

### 4.5 Qualitative subset findings

Analysis of the qualitative interviews generated three overarching themes that cut across the four interview topic areas: (1) social media as an embedded and ambivalent part of everyday student life; (2) psychological consequences shaped by patterns and interpretations of use; and (3) distress managed primarily through self-coping and trusted informal support. Importantly, experiences were heterogeneous. Some participants described upward comparison, delayed sleep, fatigue, irritability, and reduced concentration, whereas others reported little social comparison or no noticeable adverse effect on sleep or emotional wellbeing. These divergent accounts were retained because they helped clarify why duration alone may not fully represent the psychological meaning of social-media exposure. The three overarching themes and their integrated quantitative–qualitative interpretations are summarized in Table 4.

**Table 4:** Joint display of qualitative themes and quantitative findings (qualitative *n* = 12; quantitative *n* = 789).

Theme	Qualitative pattern	Representative quotation/field-note evidence	Related quantitative finding	Integrated interpretation
Embedded and ambivalent everyday use	Use throughout the day for information, entertainment, communication, academic tasks, and relief from boredom; prolonged use sometimes produced regret about lost time.	“While I am using it, I enjoy it, but when I spend too long, sometimes I feel that I have wasted my time.” (P2)	Internet/social-media use ranked fifth among perceived stressors (37.01% agreement).	Because digital engagement was normalized and often experienced positively, students may not readily identify it as a source of stress.
Psychological effects of comparison	Posts about achievement, work, travel, and lifestyles sometimes triggered pressure, inadequacy, or feeling left behind.	“When I see posts about getting a good job or traveling somewhere beautiful, I sometimes feel pressured and think that I am not good enough or that I am falling behind.” (P2)	Daily social-media duration showed the strongest adjusted association with greater CES-D severity among retained factors.	Emotional impact may depend on content and manner of engagement rather than duration alone.
Divergent response to comparison	Some participants recognized that social-media posts selectively portray positive aspects of life and resisted using them for self-evaluation.	“People usually post only the good things that happen to them…so I do not feel much pressure from other people's lives.” (P3)	The same duration category may include students with substantially different psychological experiences.	Negative/divergent cases indicate heterogeneity and caution against interpreting exposure duration as uniformly harmful.
Nighttime use and next-day functioning	Late-night scrolling delayed sleep and was followed by fatigue, irritability, mental fog, or reduced concentration; some participants reported no adverse sleep effect.	“I often use social media late at night…The next day I feel tired and mentally foggy, and my concentration in class is worse.” (P2)	>3 h/day was associated with greater depressive-symptom severity.	Sleep disruption is a plausible contextual pathway, but the cross-sectional design cannot establish temporality or causality.
Self-coping and trusted informal support	Manageable distress was handled independently; friends and family were approached when distress became harder to manage.	“When I am stressed, I usually talk to a close friend or someone in my family, or I try to manage my feelings by doing something I enjoy.” (P3)	Primary source of personal support was independently associated with CES-D severity.	Informal relationships may be perceived as more accessible and emotionally safe than formal counseling.
Barriers to professional help-seeking	Fear of judgment, privacy concerns, reluctance to burden others, and perceived lack of need could delay professional help-seeking.	Participant accounts included fear of being judged, privacy concerns, and kreng jai when considering university psychological support.	Family/close-friend support was associated with lower odds of greater symptom severity than the reference support category.	Help-seeking may reflect perceived need, trust, stigma, privacy, and cultural expectations rather than service availability alone.

**Theme 1. Social media as an embedded and ambivalent part of everyday student life.** Social media was described as a routine part of student life rather than an activity automatically identified as stressful. Participants reported using it after waking, during free periods or travel, throughout the day, and before sleep for entertainment, news and trend updates, communication, information seeking, academic tasks, and relief from boredom. Use was often experienced as enjoyable or relaxing, yet some participants described regret after prolonged use. One participant noted that social media felt enjoyable while using it but that extended use could leave a sense of having “wasted time.” This normalization helps contextualize why internet/social-media use was not among the most highly endorsed perceived stressors.

**Theme 2. Psychological consequences were shaped by patterns and interpretations of use.** Several participants described upward social comparison when viewing posts about academic or career success, travel, or desirable lifestyles, sometimes leading to pressure, inadequacy, or a sense of falling behind. One participant explained, “When I see posts about getting a good job or traveling somewhere beautiful, I sometimes feel pressured and think that I am not good enough or that I am falling behind.” However, comparison was not universal. Another participant consciously discounted online portrayals because “people usually post only the good things that happen to them,” and therefore reported little pressure from others’ lives. Nighttime use was a more consistent concern among heavier users: late scrolling delayed sleep and was followed by tiredness, irritability, mental fog, or reduced concentration the next day. Still, some participants described evening use as relaxing and reported no sleep disturbance. These contrasting accounts suggest that timing, self-regulation, content, and emotional interpretation may be as relevant as total duration.

**Theme 3. Distress was managed primarily through self-coping and trusted informal support.** Participants commonly described a stepped pattern of coping. Manageable distress was handled through self-directed strategies such as listening to music, drawing, watching series or videos, traveling, eating enjoyable food, or redirecting attention to preferred activities; social media itself could therefore function as both a potential source of strain and a coping resource. When distress became more difficult to manage, close friends and family members were commonly approached first. Professional counseling was generally viewed as a later option. One participant described reluctance to seek formal help because of fear of being judged, concern about privacy, and kreng jai (reluctance to impose on others). These accounts complement the quantitative association between primary source of personal support and depressive-symptom severity without implying a causal protective effect.

Integration of the two strands revealed both convergence and discordance. Quantitatively, longer daily social-media use was independently associated with greater depressive-symptom severity, whereas internet/social-media use ranked only fifth among perceived sources of stress. Qualitative accounts contextualized this mismatch by showing that social media was embedded in everyday academic and social routines and often served positive functions, including entertainment, information seeking, communication, and coping. At the same time, some participants described upward comparison, difficulty disengaging at night, sleep disruption, fatigue, and impaired concentration. Importantly, these experiences were not universal; several participants reported little social comparison or no perceived adverse effect on sleep or emotional wellbeing. Thus, apparently similar levels of social-media exposure may be experienced differently depending on timing, purpose, content, emotional response, and self-regulation. These findings contextualize, but do not establish causal mechanisms for, the quantitative association.

## 5 Discussion

The principal finding was not simply that daily social-media use was associated with depressive-symptom severity, but that this statistical association contrasted with students’ own ranking of internet/social-media use as only the fifth most frequently endorsed stressor. The mixed-methods design clarified this discrepancy: quantitative analyses established the pattern, while qualitative themes contextualized why social-media-related experiences may remain under-recognized. The discussion therefore focuses on this perception–association mismatch, its relation to previous evidence, its implications for university mental-health strategies, and the study's methodological limitations.

The observed association should not be interpreted as evidence that greater social-media use causes depressive symptoms. Reverse or bidirectional relationships are plausible. Prolonged or emotionally challenging online engagement may be associated with poorer psychological wellbeing, while students experiencing more severe depressive symptoms may also spend more time online for distraction, social connection, or coping. Furthermore, unmeasured factors such as loneliness, sleep disturbance, academic stress, or pre-existing psychological distress may contribute to both greater social-media use and depressive-symptom severity. Longitudinal studies with repeated assessments are needed to clarify temporal ordering and potential mechanisms.

### 5.1 Interpretation of key findings

The integrated analysis identified the perception–association mismatch as the most distinctive finding. Students most often endorsed relationship/social-environment, financial, and educational stressors, whereas internet/social-media use ranked fifth by perceived agreement. In contrast, daily social-media duration showed the strongest adjusted association with greater CES-D symptom severity among the retained regression factors. The qualitative strand added nuance by showing that digital engagement was embedded in ordinary routines and served multiple positive functions, while adverse effects clustered around particular experiences such as upward comparison, late-night scrolling, and difficulty disengaging. The presence of participants who reported little comparison or no sleep-related effect further indicates that duration alone is an incomplete indicator of psychologically relevant exposure.

- *Academic Stress*: Academic pressures are consistently linked to higher CES-D scores, indicating their significant role in student mental health (5, 19, 20, 37, 38). Students experience academic stress from pressure to maintain GPAX, rigorous coursework, competitive program demands, and concerns about future career prospects.
- *Financial Stress*: Financial difficulties are associated with elevated depressive symptoms, particularly among students experiencing financial distress (2, 29, 38, 39). Students may face financial stress from tuition fees, living expenses, and limited access to bursaries or flexible payment schemes.
- *Social Stress*: Students may experience social stress from navigating peer relationships, managing social expectations, and balancing academic demands with family obligations and campus life (40, 41). Lack of social support and discrimination stress are notable contributors to depression.
- *Lifestyle Factors*: Poor sleep hygiene, physical inactivity, and unhealthy habits often cluster together, creating cumulative physiological and psychological strain that amplifies academic and emotional pressures (5, 7, 13). These modifiable lifestyle factors can also reduce students’ coping capacity and help-seeking energy, forming a self-perpetuating cycle that worsens depressive symptoms over time.
- *Demographic and Behavioral Factors*: Gender differences, family background, and lifestyle choices (e.g., smoking, drinking) influence depression risk (5, 13, 37). Behavioral patterns including daily social-media use, reliance on teachers or others for support rather than family or close friends, and lifestyle habits like poor sleep and inactivity further compound this risk.

Stress among university students is multifaceted with various key contributors. CES-D is a reliable tool for assessing depressive symptoms, and interventions such as digital programs, positive psychology, and mindful movement have shown promising results in alleviating stress and improving mental health outcomes. Social support was also important: students who turned to family or close friends had lower odds of greater severity than those who consulted teachers or others, aligning with studies linking trusted informal support to better mental health (11, 40). However, relying on teachers or less intimate sources may signal a lack of accessible confidants or distress beyond informal management. The use of ordinal logistic regression for ordered outcomes is consistent with prior applied work and methodological guidance (52, 53). Broader evidence from Asian university populations also supports the relevance of academic, behavioral, personality, and social factors to student mental health (54–56).

### 5.2 Comparison with previous studies and the underestimated role of social media

Daily social-media use showed the strongest statistical association with depressive-symptom severity among the factors examined: 4.6% of students reporting <1 h/day were in the severe category compared with 24.1% of those reporting >3 h/day. This pattern is consistent with evidence linking heavier social-media or smartphone use with psychological distress and disrupted sleep among Asian students (3, 18, 21). The mixed-methods contribution, however, lies in the perception–association mismatch: internet/social-media use ranked only fifth among students’ perceived stressors despite its comparatively strong adjusted association with CES-D severity. Qualitative findings contextualized this discordance by showing that social media was normalized as an ordinary and often beneficial part of communication, entertainment, information seeking, academic activity, and coping, while adverse experiences clustered around particular patterns such as upward comparison, prolonged or nighttime use, sleep disruption, and difficulty disengaging. Participants who resisted comparison or reported no sleep-related effect further indicate that duration alone is an incomplete measure of psychologically relevant exposure. Source of personal support was also associated with symptom severity, and qualitative accounts showed that students commonly relied on self-coping, friends, or family before considering professional counseling, with concerns about judgment, privacy, and kreng jai shaping help-seeking. Thus, the ‘underestimated role’ refers to students’ relatively low recognition of social-media use as a stressor compared with the observed statistical association, not to evidence that social media causes depressive symptoms (3, 11, 12). Reverse or bidirectional relationships remain plausible, and longitudinal studies should incorporate richer measures of platform, activity type, timing, purpose, emotional response, and self-regulation (4, 5, 12, 18, 21). For counseling practice, assessment may therefore move beyond the single question ‘How many hours do you use social media?’ to include timing, purpose, comparison, sleep-related effects, and emotional experience.

### 5.3 Implications for AUN-HPN and university mental health strategies

Consistent with the whole-university approach underpinning health-promoting networks, these findings support embedding mental-health promotion across policy, curriculum, and student services rather than confining it to counseling alone (22, 23, 42). Routine CES-D or comparable screening should be linked to clear referral pathways, while mental-health literacy, peer-supported stress management, sleep-hygiene promotion, and digital-wellbeing education may strengthen prevention and help-seeking (11–13, 21, 43, 44). Structural supports, including bursaries and flexible payment options, should complement clinical services for students experiencing financial strain (5, 38). Evidence-based approaches for university students include mindfulness-based and cognitive-behavioral interventions, positive psychology, creative or physical activities, mind-body practices, and scalable self-guided or chatbot-supported digital programs (10, 38, 44–50). Given that no single modality suits all students, a layered strategy combining informal support networks, culturally responsive counseling, digital tools, academic-risk monitoring, and referral pathways is most consistent with the multifactorial pattern observed in this study. Embedding these components within the AUN-HPN framework could help universities move from prevalence documentation toward coordinated and transferable mental-health promotion.

### 5.4 Practical recommendations for university counseling centers

Based on the findings presented in Section 4, the following recommendations may help university counseling centers strengthen early identification, targeted support, and culturally responsive services for students reporting depressive symptoms:

- *Routine mental health screening*: CES-D or comparable screening should be integrated into student health services, particularly for students with academic risk markers such as senior-year standing, lower GPAX, and heavy daily social-media use. Screening must be linked to clear referral pathways rather than functioning only as data collection.
- *Digital-wellbeing assessment on intake forms:* Counseling intake forms may include questions about passive scrolling, social comparison, nighttime use, emotional responses after online engagement, and perceived difficulty disconnecting (4, 18, 43, 44). These questions may help counselors identify patterns of digital engagement associated with depressive symptoms.
- *Digital-wellbeing workshops*: Workshops should be offered through general education or student development programs, focusing on self-awareness, healthy digital boundaries, sleep hygiene, emotional regulation, and coping strategies, rather than simply warning students to reduce screen time (19, 38, 43).
- *Peer-support and family-informed programs*: Because confiding in family or close friends was associated with lower odds of greater symptom severity, peer gatekeepers, faculty advisors, and student leaders may be trained to recognize signs of distress, respond supportively, and facilitate referral when needed. This association should not be interpreted as evidence of a causal protective effect (11, 40).
- *Culturally responsive mental health promotion*: Messages should reduce stigma, normalize help-seeking, and address Thai cultural concerns such as face-saving, respect for hierarchy, academic expectations, and reluctance to disclose emotional distress (5, 9, 51). Counseling centers should promote help-seeking as a strength and position mental health support as part of student success.

### 5.5 Limitations and methodological considerations

Several limitations should be considered. First, the cross-sectional design precludes causal conclusions and does not establish temporal ordering. The relationship between social-media use and depressive symptoms may be bidirectional, and residual confounding by sleep quality, loneliness, academic stress, or pre-existing psychological distress cannot be excluded. Second, social-media use was measured only by daily duration; the qualitative findings themselves demonstrate that similar duration may encompass very different experiences, supporting future assessment of platform, activity type, timing, purpose, emotional response, and self-regulation. Third, depressive symptoms were measured using the CES-D, a screening instrument rather than a diagnostic interview. Fourth, convenience sampling at one private Thai university limits generalizability and may introduce selection bias. Fifth, the qualitative subsample was small, and data were based primarily on contemporaneous field notes rather than full verbatim transcripts, which may have reduced interpretive depth and limited the availability of direct quotations. Although thematic sufficiency was assessed and two additional interviews were conducted after the tenth interview, larger and more diverse qualitative samples would strengthen transferability. Finally, mixed-methods integration provides contextual interpretation but cannot verify causal mechanisms.

## 6 Conclusion

This mixed-methods study found that 49.68% of 789 undergraduates reported clinically significant depressive-symptom scores (CES-D ≥ 16). Its principal contribution is the discrepancy between perceived and observed patterns: internet/social-media use ranked only fifth among eight perceived stressors (37.01% agreement), yet daily use showed the strongest adjusted association with depressive-symptom severity among the factors examined. The qualitative strand showed why this relationship may be under-recognized: social media was embedded in everyday student life and often served positive functions, while adverse experiences varied according to upward comparison, nighttime use, sleep disruption, and self-regulation. Importantly, some students reported little comparison or no adverse sleep effects, underscoring that duration alone does not capture the psychological meaning of digital exposure. Students also tended to manage distress through self-coping and trusted informal relationships before considering professional support. Integrating the two strands therefore provided a fuller interpretation than either could provide alone. The findings support digital-wellbeing assessment that considers timing, purpose, content, emotional response, and self-regulation alongside routine mental-health screening, sleep-hygiene promotion, peer and family support, and culturally responsive counseling. Because the study was cross-sectional, the findings identify an under-recognized risk marker rather than a causal effect. Longitudinal, multi-institutional studies using richer measures of digital engagement are needed to establish temporal ordering and test the proposed pathways.

## Data Availability

The original contributions presented in the study are included in the article/Supplementary Material; further inquiries can be directed to the corresponding author.
